# Recurrent intracranial hemorrhage secondary to congenital factor XIII deficiency: A case report and literature review

**DOI:** 10.1097/MD.0000000000047969

**Published:** 2026-03-27

**Authors:** Xu Zichao, Xu Wei, Yu Hai, Wu Jianyue

**Affiliations:** a Hangzhou Normal University, Hangzhou, Zhejiang, China.

**Keywords:** congenital FXIII deficiency, intracerebral hemorrhage

## Abstract

**Rationale:**

Coagulation factor deficiencies can lead to hemorrhagic syndromes of varying severity and bleeding manifestations. Intracranial hemorrhage (ICH) associated with coagulation factor XIII (FXIII) deficiency is particularly challenging because its clinical presentation closely mimics spontaneous ICH, often occurring without apparent triggers. Routine coagulation tests (prothrombin time and activated partial thromboplastin time) are typically normal, contributing to diagnostic difficulties and frequent underdiagnosis or misclassification. As the management of this condition differs from other ICH types, replacement therapy with cryoprecipitate or fresh frozen plasma is crucial beyond surgical intervention. This case report highlights this rare, life-threatening condition to enhance clinical recognition.

**Patient concerns:**

A 53-year-old Chinese male presented with recurrent ICH over a 20-day period and underwent 2 intracerebral hematoma evacuation surgeries. Postoperative head computed tomography (CT) revealed persistent rebleeding at the site of original hemorrhage.

**Diagnoses:**

Congenital FXIII deficiency, intracerebral hemorrhage, and cerebral herniation.

**Interventions:**

The patient underwent surgical hematoma evacuation followed by allogeneic blood transfusion therapy, and received 10 units of cryoprecipitate daily.

**Outcomes:**

After 35 days of treatment, the patient regained full consciousness with normal cognitive and neurological responsiveness. Follow-up CT imaging revealed significant resolution of the hematoma. He was discharged in stable condition. Post-discharge management included weekly infusions of 6 to 10 units of cryoprecipitate at local medical facilities along with routine monitoring of coagulation profiles and cranial CT scans.

**Lessons:**

Intracranial hemorrhage secondary to FXIII deficiency is exceedingly rare. Clinicians should consider FXIII deficiency when recurrent ICH occurs with normal routine coagulation tests, to enable early diagnosis and life-saving replacement therapy. Early identification of the underlying etiology through comprehensive diagnostic evaluation is essential to guide effective and timely interventions, particularly in the context of increasing ICH incidence and healthcare resource utilization.

## 1. Introduction

Coagulation factor XIII (FXIII) deficiency is exceptionally rare. It exists in both congenital and acquired forms, with the congenital type having an estimated incidence of approximately one in 2 to 3 million individuals. To date, only slightly over 100 cases have been documented in China, a scarcity that likely contributes to frequent misdiagnosis.^[1]^ Diagnosis is challenging because routine coagulation tests are typically normal, necessitating specific confirmatory tests such as the urea solubility test, FXIII activity assay, or genetic analysis.

The cause of congenital FXIII deficiency may be mutations in the F13A or F13B genes. Mutations in the A subunit are more common and usually cause more severe phenotypes.^[2,3]^ The hallmark of FXIII deficiency is delayed bleeding, with commonly affected sites including the intracranial space, umbilical cord, soft tissues (e.g., subcutaneous and muscular bleeding), postoperative wounds, nasal mucosa (epistaxis), and gastrointestinal tract.

Intracranial hemorrhage(ICH) accounts for approximately 25 to 30% of cases and is the leading cause of mortality in patients with congenital FXIII deficiency.^[4,5]^ Herein, we present a rare case of recurrent ICH secondary to congenital FXIII deficiency, to enhance clinical awareness and underscore the critical importance of early diagnosis and management. FXIII deficiency should be included in the differential diagnosis for patients presenting with unexplained ICH.

## 2. Case presentation

**Figure 1.**
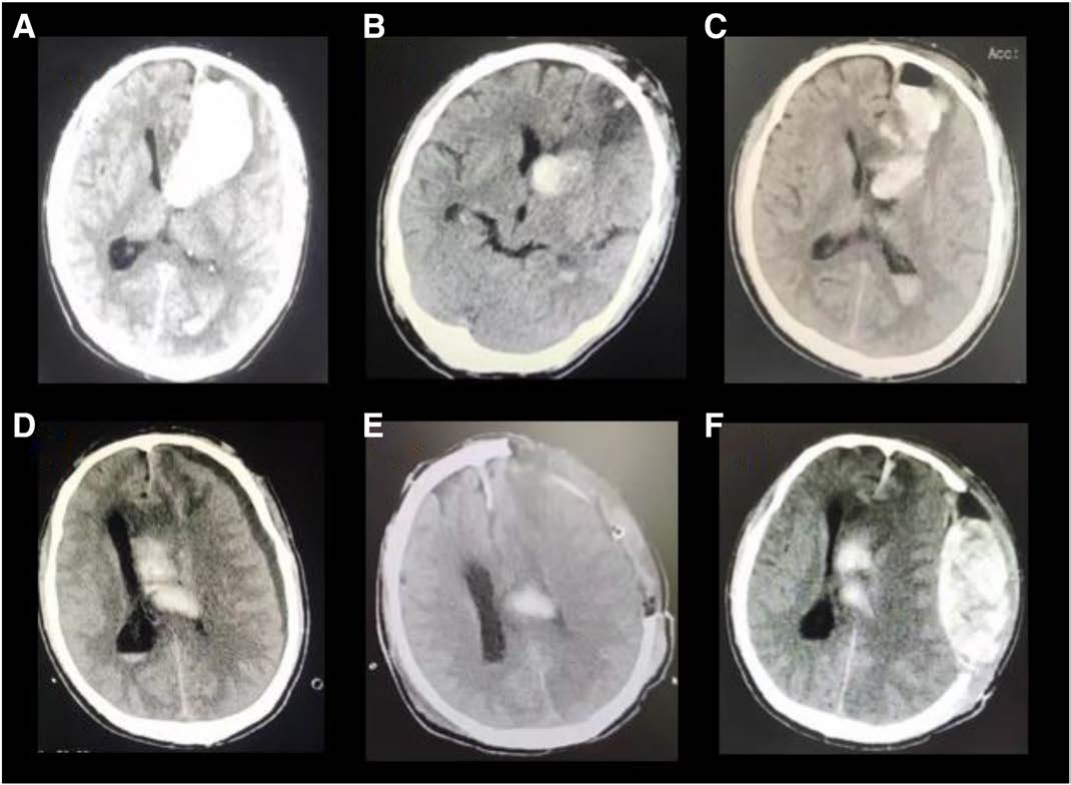
Cranial CT imaging from the referring hospital. (A) Day 1: Left frontotemporal lobe hemorrhage on admission. This explains the patient dizziness and headache (B) Post-first surgery follow-up scan: Reduced hematoma volume in the left frontotemporal region. The patient consciousness has improved. (C) Postoperative day 4 follow-up scan: New-onset hemorrhage in the left frontotemporal lobe. The patient consciousness is blurred again. (D) Postoperative day 7 follow-up scan: Left lateral ventricular hemorrhage with left subdural effusion. (E) Post-second surgery follow-up scan: Decreased intraventricular hemorrhage volume compared to prior imaging. (F) Post-second surgery day 4 follow-up scan: Left frontotemporal epidural and subdural hematomas causing midline shift due to mass effect from the hematoma. The patient is unconscious. CT = computed tomography.

A 53-year-old male was admitted to our hospital in June 2023 for the evaluation and treatment of recurrent ICH. The patient had no relevant medical history. Twenty days prior to referral, he experienced sudden onset of dizziness and headache. Initial cranial computed tomography (CT) performed at a local hospital revealed a large left frontotemporal hemorrhage (Fig. 1A) with an estimated hematoma volume exceeding 30 mL, warranting emergency surgical intervention. He underwent prompt endoscopic hematoma evacuation.

A follow-up CT scan on postoperative day 2 (Fig. 1B) showed a reduction in the hematoma volume within the left frontotemporal region. However, on postoperative day 4, the patient developed altered mental status. Repeat imaging revealed a new hemorrhage at the original site (Fig. 1C) for which conservative management was initiated. Three days later, CT demonstrated intraventricular hemorrhage in the left lateral ventricle along with left-sided subdural effusion (Fig. 1D), necessitating a second surgical procedure, hematoma evacuation, combined with decompressive craniectomy. Postoperative imaging (Fig. 1E) showed partial resolution of the intraventricular hemorrhage and a left-sided craniectomy defect.

Despite these interventions, the patient remained comatose. A subsequent CT scan obtained 3 days later revealed epidural and subdural hematomas in the left frontotemporal region, with an associated midline shift due to the mass effect (Fig. 1F). The patient was transferred to our hospital for further care.

**Figure 2.**
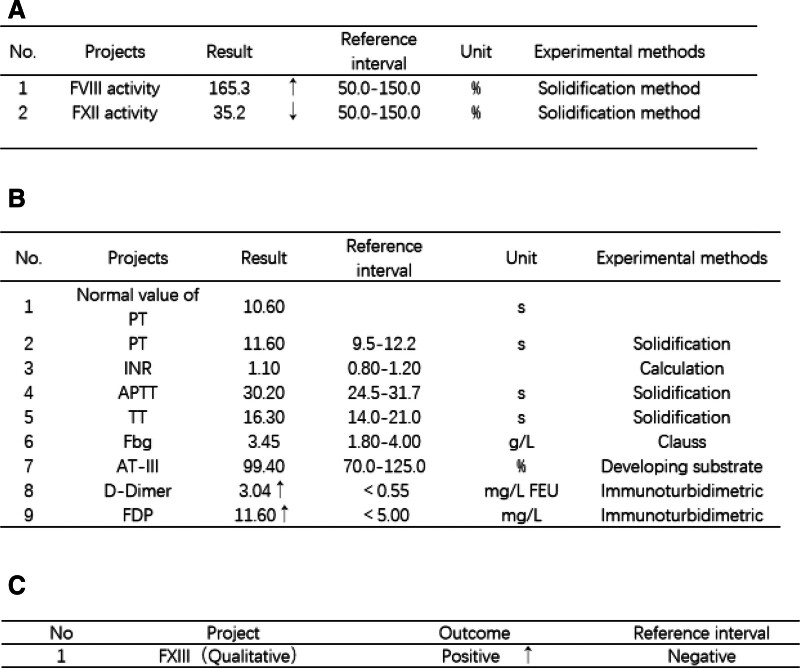
Admission laboratory findings. (A) Routine coagulation factor assays: Only FXII activity was decreased. (B) Coagulation function tests: No significant abnormalities detected. (C) Urea solubility test: Positive result, confirming FXIII deficiency. APTT = activated partial thromboplastin time, AT-III = antithrombin III, Fbg = fasting blood glucose, FDP = fibrin/fibrinogen degradation products, FEU = fibrinogen equivalent units, FVIII = factor VIII, FXII = factor XII, FXIII = factor XIII, INR = International normalized ratio, PT = prothrombin time, TT = thrombin time.

Upon admission to our hospital, the patient presented with stable vital signs: blood pressure of 128/87 mm Hg and a body temperature of 37.5°C. Pupillary examination revealed equal and reactive pupils, each with a diameter of 3 mm. Neurological assessment demonstrated muscle strength of grade 3 in all 4 limbs, bilaterally negative Babinski signs, and positive neck stiffness. Initial laboratory investigations revealed leukocytosis, with a white blood cell count of 13.60 × 10^9^/L. The cerebrospinal fluid appeared turbid and xanthochromic. Analysis revealed a nucleated cell count of 213/L, glucose at 1.8 mmol/L, and lactate at 6.2 mmol/L. Based on these physical and laboratory findings, a diagnosis of postoperative intracranial infection was made, and empirical intravenous vancomycin therapy was initiated. Repeat lumbar puncture after 3 days revealed a nucleated cell count of 85/μL, glucose at 2.4 mmol/L, and lactate at 1.5 mmol/L, indicating a significant resolution of the infection. To identify the cause of recurrent hemorrhage, we performed an extensive coagulation workup. Coagulation factor assays (Fig. 2A) and corresponding functional tests (Fig. 2B) revealed only mildly reduced factor XII activity. However, as factor XII deficiency is not typically associated with clinically significant bleeding, this abnormality was considered unrelated to the current hemorrhagic events.

**Figure 3.**
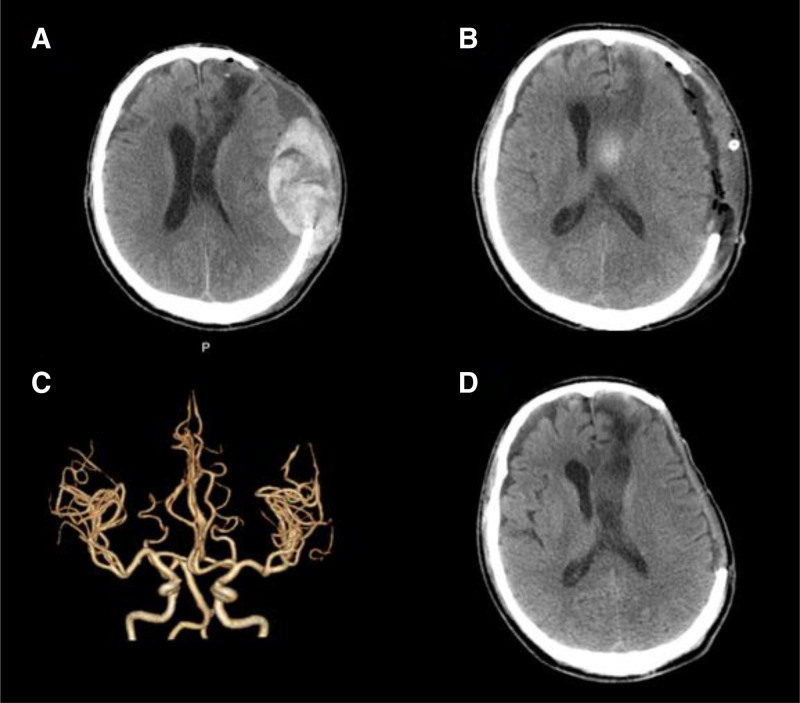
Admission and follow-up neuroimaging. (A) Cranial CT on hospital day 4: Progression of left frontotemporoparietal subdural/epidural hematomas with midline shift to the right and postoperative left cranial bone defect. The patient is currently in a coma. (B) Cranial CT on postoperative day 1: Partial resolution of left frontotemporoparietal subdural/epidural hematomas; residual hemorrhage in the left lateral ventricle, persistent midline shift, and postoperative cranial defect. (C) CTA: No significant stenosis or dilation observed in the lumens of intracranial arteries. (D) Final follow-up cranial CT: No evidence of rebleeding; minimal residual effusion in the left frontotemporal region with persistent postoperative cranial defect. Following discharge, the patient maintained clear consciousness with no reports of dizziness or headache. CT = computed tomography, CTA = computed tomography angiography.

Four days after admission, the patient experienced a decline in consciousne**ss**. Repeat cranial CT (Fig. 3A) revealed progression of the subdural and epidural hematomas in the left frontotemporal and frontoparietal regions, accompanied by increased fluid accumulation. Emergency evacuation of the intracerebral hematoma was performed, and the patient was subsequently transferred to the intensive care unit for postoperative monitoring. Postoperatively, the patient neurological status improved, with regained responsiveness to verbal stimuli. Within 1 week, serial cranial CT scans (Fig. 3B) demonstrated gradual absorption of residual hemorrhage in the left lateral and third ventricles, although a small amount of blood remained.

On postoperative day 10, the patient consciousness deteriorated for a second time. Given the recurrence of hemorrhage despite multiple surgical interventions, we initiated a systematic evaluation to identify the underlying etiology. The diagnostic algorithm proceeded as follows. First, computed tomography angiography (Fig. 3C) was performed, which ruled out congenital vascular anomalies like aneurysms or arteriovenous malformations. Second, cerebral amyloid angiopathy was considered highly unlikely given the patient relatively young age and the hematoma distribution (including epidural involvement), which is uncharacteristic of this pathology. After excluding these structural and common acquired causes, the recurrent bleeding tendency strongly suggested an underlying coagulopathy, raising specific suspicion for a coagulation factor deficiency.

According to our hospital standards, if the blood clot dissolves within 1 hour, it is considered positive. A qualitative FXIII assay (urea solubility test) (Fig. 2C) yielded a positive result, confirming FXIII deficiency as the underlying cause of recurrent hemorrhage. All relevant test results are summarized in Table 1. To investigate the genetic basis, peripheral blood samples from the patient and his parents underwent whole exome sequencing at Hangzhou Daan Medical Laboratory. No genetic abnormalities were detected. Therefore, further genetic testing was recommended to identify the underlying cause. However, the patient and the family declined additional testing due to personal reasons. In the absence of comorbidities or suggestive drug history, a diagnosis of congenital FXIII deficiency was established. According to the Guidelines for the Management of Acute Hemorrhagic Coagulopathy, allogeneic transfusion therapy consisting of daily infusions of 10 units of cryoprecipitate was initiated immediately. Follow-up CT showed no further bleeding, and the patient regained full consciousness with normal verbal responsiveness.

**Table 1:** Summary of results.

S. No.	Projects	Result	Reference interval	Unit
1	Normal value of PT	10.60		s
2	APTT	30.20	24.5–31.7	s
3	Fbg	3.45	1.80–4.00	g/L
4	FXIII (Qualitative)	Positive↑	Negative	
5	PLA	234	100–300	g/L
6	CTA	Normal	Normal	

APTT = activated partial thromboplastin time, CTA = computed tomography angiography, Fbg = fasting blood glucose, FXIII = coagulated factor XIII, g/L = gram per litre, PT = prothrombin time, PLA = polylactic acid, s = second.

**Figure 4.**
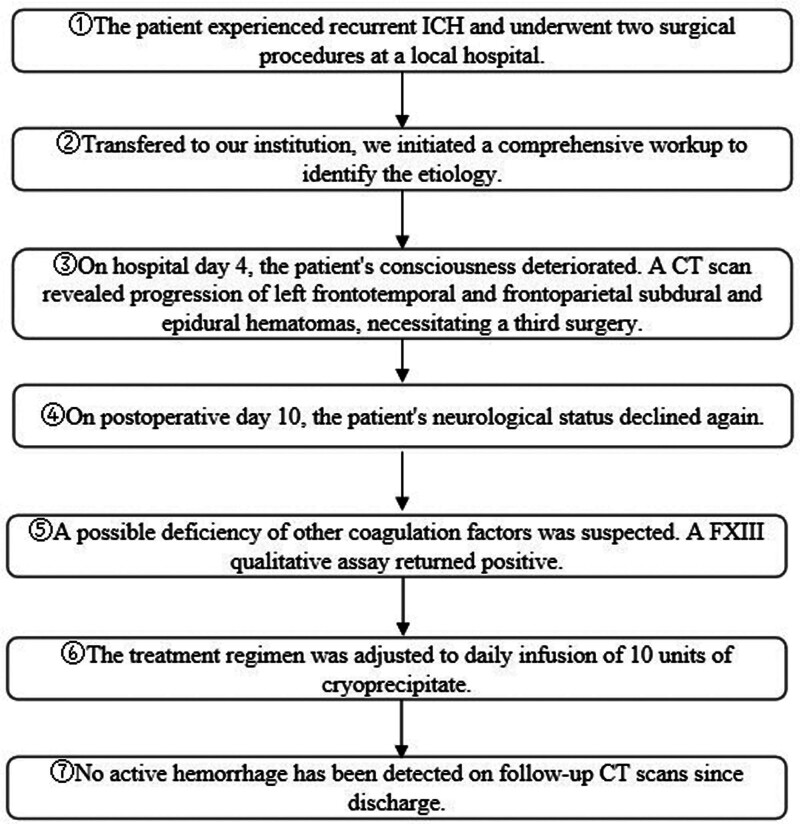
Patient diagnosis and treatment timeline chart.

Due to concurrent intracranial infection, cranioplasty was contraindicated, and a delay of at least 1 year was recommended. After discharge, the patient received weekly infusions of 6 to 10 units of cryoprecipitate at local hospitals along with regular monitoring of coagulation parameters and cranial imaging. During 12 months of follow-up, the patient remained alert, with no radiographic evidence of recurrent hemorrhage (Fig. 3D). Although cranioplasty has not yet been performed, the intracranial cavity appears stable. Upon follow-up, the patient remained free of dizziness and headache and evaluated the clinical management as satisfactory. He has continued maintenance cryoprecipitate therapy at our institution and is currently being evaluated for future cranioplasty. The complete timeline of this diagnosis and treatment of the disease will be presented below (Fig. 4).

## 3. Discussion

In this case, acute management (including surgical hematoma evacuation and antimicrobial therapy) was essential to arrest disease progression and stabilize the patient. Simultaneously, a comprehensive etiological investigation extending beyond standard coagulation screening ultimately identified FXIII deficiency using a qualitative urea solubility test. Additional laboratory techniques available for diagnosing FXIII deficiency include biotin-based activity assays and ELISA-based antigen quantifications. The urea solubility test, which is one of the most widely used screening methods, involves incubating patient plasma with calcium chloride (Ca^2+^) to facilitate the conversion of fibrinogen into a fibrins clot. The clot was then immersed in a 5 mol/L urea solution; in FXIII-deficient plasma, the clot rapidly dissolved due to impaired cross-linking, confirming the deficiency.^[2,6]^ The diagnosis can be established by a FXIII qualitative assay, with subsequent genetic testing to differentiate congenital from acquired disease. In our patient, whole exome sequencing of the patient and family members detected no pathogenic variants in the coding regions of *F13A1* or *F13B*. This negative result may be attributed to the analysis being limited to coding regions, excluding potential mutations in regulatory noncoding regions. We recommended further genetic testing with an expanded panel. However, the patient declined a second round of testing for personal reasons.

Early recognition of FXIII deficiency and timely initiation of prophylactic therapy are essential for preventing life-threatening bleeding events and mitigating the risk of severe neurological sequelae.

In addition to surgical control of active bleeding, replacement therapy with fresh frozen plasma (FFP), cryoprecipitate, or FXIII concentrate is essential. In settings where FXIII concentrate is unavailable, cryoprecipitate serves as an effective alternative due to its FXIII content. In China, cryoprecipitate and FFP are the standard treatment modalities, whereas internationally, FXIII concentrates derived from placental extracts or plasma are also used. In this case, the patient received cryoprecipitate as replacement therapy.

According to Article XIII of the Expert Consensus on Hereditary Coagulation Disorders, the recommended dose of cryoprecipitate for FXIII deficiency is 1 unit per 10 kg of body weight.^[7]^ In emergency settings, where FFP, cryoprecipitate, or FXIII concentrate is unavailable, platelet transfusion may serve as an alternative, as FXIII is stored in platelet α-granules. Replacement therapy forms the cornerstone of long-term management in FXIII deficiency, typically entailing lifelong treatment to maintain effective prophylaxis against bleeding.

In cases of suspected FXIII deficiency, neurosurgeons must leverage a combination of diagnostic tools such as qualitative assays, activity tests, ELISA, and genetic testing, to accurately identify the disorder.

**Figure 5.**
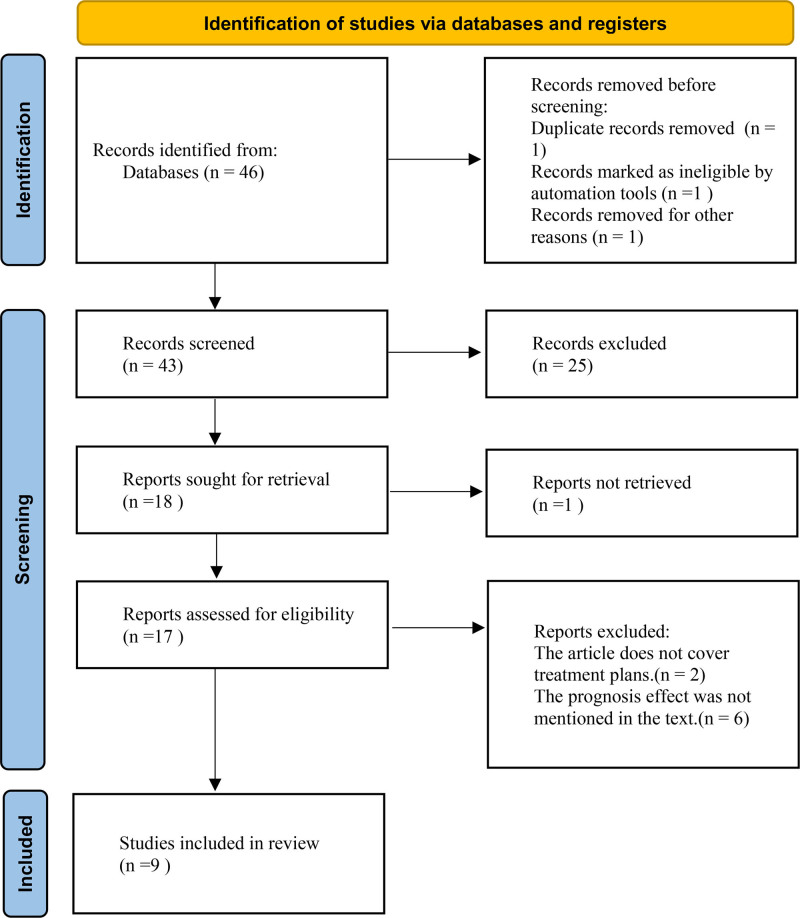
Flowchart.

Through literature retrieval, we identified 46 articles on congenital FXIII deficiency. Based on our inclusion criteria: inclusion of cases with ICH due to congenital FXIII deficiency, clearly documented treatment regimens, and availability of posttreatment outcomes: 9 articles were ultimately included as relevant for analysis. The flow diagram is presented in Figure 5.

A review of 9 case reports encompassing 73 patients with congenital FXIII deficiency revealed that ICH occurred in 17 cases (23.3%).^[1,5,8–14]^ This incidence aligns with previously cited global reports. The basic demographic characteristics of these 17 ICH cases are summarized in Table 2, showing a female predominance (10 cases, 58.8%) over males (7 cases, 41.2%) and a mean age of 38.8 years at first hemorrhage. All the affected individuals received long-term plasma-derived replacement therapy to prevent recurrence. Notably, 3 reports explicitly described a two-phase management strategy, comprising initial surgical intervention for ICH followed by lifelong prophylactic replacement therapy. Based on our series of 17 cases, we found that ICH in the setting of congenital FXIII deficiency consistently necessitates treatment. Therefore, we recommend initiating immediate empirical replacement therapy with plasma products for all patients presenting with this condition.

**Table 2:** Patient characteristics.

Patient	Gender	Age (yr)	Treatment regimen	Therapies	Result
1	F	48	ODT then LTP	surgery and cryoprecipitate	Rehabilitation
2	F	63	LTP	Cryoprecipitate	Rehabilitation
3	M	84	LTP	FFP	Rehabilitation
4	M	1	LTP	FFP	Rehabilitation
5	F	26	LTP	FFP	Rehabilitation
6	M	53	LTP	FFP	Rehabilitation
7	M	7	LTP	Cryoprecipitate	Rehabilitation
8	F	76	LTP	FFP	Rehabilitation
9	F	98	LTP	FFP	Rehabilitation
10	F	9	LTP	Cryoprecipitate	Rehabilitation
11	F	1	LTP	Cryoprecipitate	Rehabilitation
12	F	41	LTP	Cryoprecipitate	Rehabilitation
13	M	62	LTP	FFP	Rehabilitation
14	M	22	LTP	FFP	Rehabilitation
15	F	62	LTP	Cryoprecipitate	Rehabilitation
16	F	4	ODT then LTP	surgery and FFP	Rehabilitation
17	M	2	ODT then LTP	surgery and FFP	Rehabilitation

F = female, FFP = fresh frozen plasma, LTP = long-term prophylaxis, M = male, ODT = on demand treatment.

## 4. Conclusion

From a diagnostic perspective, patients with congenital FXIII deficiency typically present with normal prothrombin time, activated partial thromboplastin time, and thrombin time, which may lead to under-recognition or misdiagnosis. Clinically, bleeding can occur at various anatomical sites, including the face, trunk, extremities, and intracranial cavity, further complicating diagnosis. Important differential diagnoses include hemophilia A and B, von Willebrand disease, and disseminated intravascular coagulation. Clinicians should maintain a high index of suspicion for FXIII deficiency in patients presenting with single or multiple bleeding sites, particularly those with ICH. This case highlights the importance of suspecting FXIII deficiency in unexplained ICH with normal coagulation screens. Early diagnosis and prompt replacement therapy can be lifesaving. From a therapeutic standpoint, for patients with clear surgical indications such as mass effect or progressive neurological deterioration: prompt surgical intervention is essential for the management of acute hemorrhage. Postoperatively, long-term replacement therapy with cryoprecipitate or FFP is necessary to reduce the risk of recurrent bleeding and ensure sustained hemostatic stability.

## Acknowledgments

We would like to thank the patients and their families for allowing us to use their clinical data in this study.

## Author contributions

**Formal analysis:** Xu Zichao, Xu Wei, Yu Hai.

**Funding acquisition:** Xu Zichao, Yu Hai.

**Writing – original draft:** Xu Zichao.

**Writing – review & editing:** Wu Jianyue, Yu Hai.
